# Current and Emerging Methods of Antibiotic Susceptibility Testing

**DOI:** 10.3390/diagnostics9020049

**Published:** 2019-05-03

**Authors:** Zeeshan A. Khan, Mohd F. Siddiqui, Seungkyung Park

**Affiliations:** School of Mechanical Engineering, Korea University of Technology and Education, Cheonan, Chungnam 31253, Korea; iamzak@koreatech.ac.kr (Z.A.K.); mohdfarhan@koreatech.ac.kr (M.F.S.)

**Keywords:** Antibiotic susceptibility tests, resistance, phenotypic, genotypic, bacteria

## Abstract

Antibiotic susceptibility testing (AST) specifies effective antibiotic dosage and formulates a profile of empirical therapy for the proper management of an individual patient’s health against deadly infections. Therefore, rapid diagnostic plays a pivotal role in the treatment of bacterial infection. In this article, the authors review the socio-economic burden and emergence of antibiotic resistance. An overview of the phenotypic, genotypic, and emerging techniques for AST has been provided and discussed, highlighting the advantages and limitations of each. The historical perspective on conventional methods that have paved the way for modern AST like disk diffusion, Epsilometer test (Etest), and microdilution, is presented. Several emerging methods, such as microfluidic-based optical and electrochemical AST have been critically evaluated. Finally, the challenges related with AST and its outlook in the future are presented.

## 1. Introduction

Antibiotic resistance is defined as the genetic ability of bacteria to encode the resistance genes that counterfeit the inhibitory effect of potential antibiotics for survival [1]. It can be developed either intrinsically by natural recombination and integration into the bacterial genome, or it can be acquired through horizontal gene mutation events such as conjugation, transformation, and transduction [2]. The prominent events in the generation of bacterial resistance include inactivation of the porin channel, modification of antibiotic targets, and neutralizing antibiotic efficacy through enzymatic action [3]. Thus, the understanding of the genetic makeover and the morpho-anatomical changes in bacteria are of prime importance to counteracting the resistance mechanism.

The discovery of antibiotics was paradigm-altering, as it was not only an effective tool against chronic infections but also opened new avenues for drug industries. Global antibiotic statistics suggested an increase of 35% in the antibiotic consumption between 2000 and 2010, and the current antibiotic industry stands at USD 39.8 billion (up to 2015). Russia, India, China, Brazil, and South Africa are major contributing countries, where 76% of the rise in antibiotic consumption has been estimated [4]. The changes experienced in the enhanced consumption of antibiotics over the past decade remain unprecedented, and this is chiefly a result of the emergence of new diseases. Alternately, the increase in antibiotic consumption and industrialization might be due to overuse or misuse of antibiotics recommended by physicians/self-medication at the time of infection [5,6]. A recent report on the casualties related to antibiotic resistance by the world health organization (WHO) depicted an alarming 700,000 lives per year currently, and predicts a disturbing 10 million/year by 2050, ensuring that antibiotic resistance will be the most prevalent cause of death [7]. Adding to this, WHO also forewarns the severity of antibiotic resistance, stating that “it threatens the achievements of modern medicine, a post-antibiotic era—in which common infections and minor injuries can kill—is a very real possibility for the 21st century” [8].

Minimum inhibitory concentrations (MICs) of various antimicrobial susceptibility testing (AST) are categorized by various international agencies. These MIC guidelines determine whether an antibiotic is susceptible or not. The Clinical and Laboratory Standards Institute (CLSI) provides the most popular guidelines, which are based on pharmacokinetic–pharmacodynamic (PK-PD) properties and mechanisms of resistance [9]. Most European countries follow the MIC cut-offs based on PK-PD properties, and the epidemiological MIC cut-offs (ECOFFS) as determined by the European Committee on Antimicrobial Susceptibility Testing (EUCAST). The MIC breakpoints recommended by EUCAST are generally higher than the CLSI. Because of the modifications in the guidelines, the results are substantially changed, such as higher ceftazidime resistance in *Klebsiella pneumonia* and ESBL-producing *Escherichia coli* (*E. coli*). Moreover, the CLSI guidelines re accessible for non-members as a package of three documents for USD 500 annually, while EUCAST guidelines are freely available on the EUCAST website [9]. 

**Figure 1:**
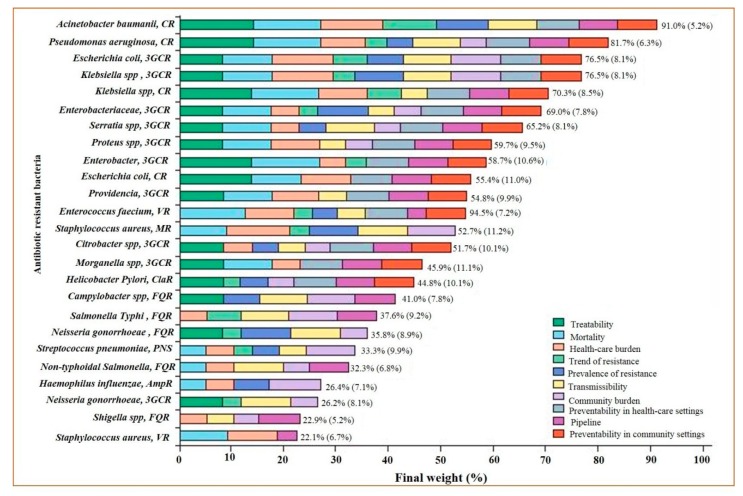
Representation of the final ranking of antibiotic-resistant bacteria, adapted with permission from Tacconelli et al. [11]. CR = carbapenem resistant. 3GCR = third-generation cephalosporin resistant. VR = vancomycin resistant. MR = meticillin resistant. ClaR = clarithromycin resistant. FQR = fluoroquinolone resistant. PNS = penicillin non-susceptible. AmpR = ampicillin resistant.

A sharp surge in bacteria-encoding resistance is occurring worldwide, jeopardizing the efficacy of antibiotics that have saved millions of lives [10]. The antibiotics which have threatened bacteria for decades are under grave threat themselves. Owing to large societal repercussions of multidrug resistance and the significantly reduced development of drugs, it is mandatory to determine the microbes which need more attention than others for drug development. Consequently, WHO has developed a priority list of the pathogens, and stratified the list into critical, high, and medium priorities. Carbapenem-resistant *Pseudomonas aeruginosa* and *Acinetobacter baumanii,* carbapenem-resistant and third-generation cephalosporin-resistant *Enterobacteriaceae* were placed in critical-priority bacteria. The high priority bacteria included vancomycin-resistant *Enterococcus faecium*, methicillin-resistant *Staphylococcus aureus* (MRSA), clarithromycin-resistant *Helicobacter pylori*, fluoroquinolone-resistant *Campylobacter* spp., penicillin-resistant *Streptococcus pneumoniae*, ampicillin-resistant *Haemophilus influenzae*, and fluoroquinolone-resistant *Shigella* spp. [11] (Figure 1). Resistance against beta-lactam antibiotics like penicillin is widespread, while resistance against other drugs, such as vancomycin and fluoroquinolone, are less frequently observed (Supplementary Table S1). The infections arising from resistant bacteria, because of mutations, might present themselves with harsher symptoms than their predecessors (Supplementary Table S1). Although novel drugs have shown much promise against these resistant bacteria, their rapid diagnostic is still a huge concern (Supplementary Table S1). 

In this review, we concisely discuss the advantages and limitations of various tools for AST (Supplementary Table S2). We first review the phenotypic methods for AST like diffusion, dilution, and automated AST tools. Central aspects of genotype-based AST methods, including susceptibility diagnostics based by polymerase chain reaction (PCR) and DNA microarray, are addressed. Then, an overview of several emerging approaches such as fluorescent, colorimetric, and electrochemical microfluidic sensors, with their related caveats, are discussed (Supplementary Table S2).

## 2. Phenotypic AST Methods

### 2.1. Diffusion 

**Figure 2:**
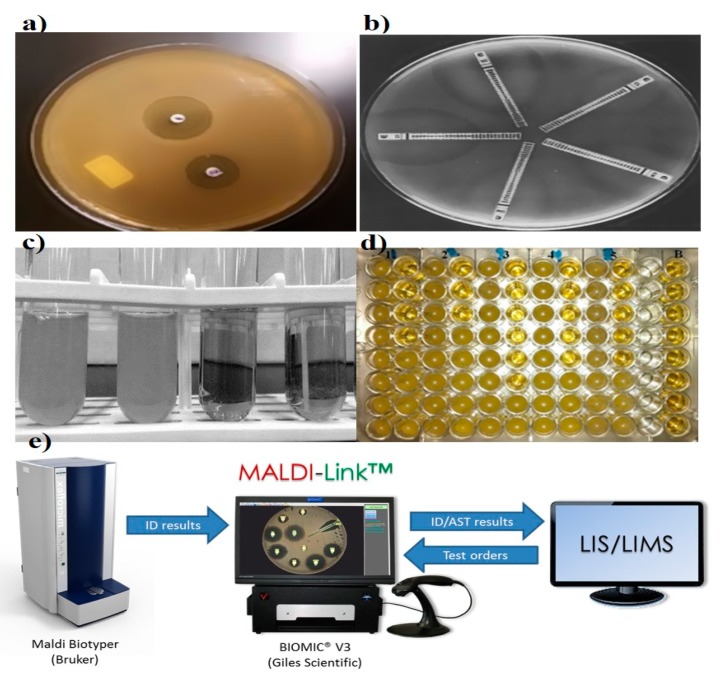
Representation of various conventional antibiotic susceptibility testing methods. (**a**) Disk diffusion, demonstrating of inhibition zones, adapted from Sageerabanoo [29]. (**b**) Etest gradient disk diffusion, adapted from Sader [30], under terms of the Creative Commons attribution license. (**c**,**d**) Broth macro and micro dilution, showing bacterial susceptibility based on optical density and (**e**) Matrix-Assisted Laser Desorption Ionization-Time of Flight Mass Spectrometry (MADI-TOF MS), adapted from the MALDI Biotyper system (Bruker, Billerica, Massachusetts, United States), Laboratory Information System (LIS) and Laboratory Information Management System (LIMS).

The disk diffusion method is the gold standard for confirming the susceptibility of bacteria. Standardized disk diffusion was introduced by Bauer and Kirby’s experiments in 1956, after finalizing all aspects of optimization by changing physical conditions [12]. In this method, the isolated bacterial colony is selected, suspended into growth media, and standardized through a turbidity test. The standardized suspension is then inoculated onto the solidified agar plate, and the antibiotic-treated paper is tapped on the inoculated plate. The disc containing the antibiotic is allowed to diffuse through the solidified agar, resulting in the formation of an inhibition zone after the overnight incubation at 35 °C. Thereafter, the size of the inhibition zone formed around the paper disc is measured; the size of the inhibition zone corresponds to the concentration of antibiotic (Figure 2) [12,13]. Assessing and determining the susceptibility of bacteria generally takes 16–24 h. Several diffusion-based experiments have been performed prior to the standardized disk diffusion method. In the 1920s, Fleming was the pioneering contributor to AST. Fleming’s gutter method was the first method of antibiotic analysis where antibiotic was dispensed into a gutter made on solid agar that allowed the antibiotics to diffuse through it [14]. A modification to this design, called the “Oxford cup method,” was subsequently developed by Abraham et al. in 1941, where the gutter was replaced with a glass cup for diffusion [15]. Simultaneously, in the 1940s, Pope (1940), Foster and Woodruff (1943), and Vincet and Vincet (1944) used an antibiotic-impregnated paper disc for the diffusion of antibiotics [16,17]. These methods were hindered by inaccurate analysis due to evaporation, difficulty in handling, sterilization, and cumbersome operation [18]. Moreover, a single antibiotic was focused on susceptibility testing (i.e., penicillin). In later years, the introduction of effective drugs and convenient means of susceptibility testing have prevailed with the increase of the deadly infections. Therefore, variations of the method have been adopted to expand its versatility and utility. In 1947, Hoyt, Levine, and Bondi introduced penicillin tablets and the standard 6.5-mm disk method separately to emphasize multiple targets [19,20]. In the 1950s, experiments by Gould and Bowie (1952) and Stokes (1955) enabled the differentiation between susceptible and resistant bacteria through the multiple disk diffusion technique [21,22]. All the proposed methods were inaccurate, unsuitable, and unreliable for routine testing because of discrepancies in results obtained from different labs [23]. Hence, in 1961, several organizations (especially WHO) made several efforts to address the need for a standardized method for antibiotic susceptibility testing. Later, in the year 1966, Bauer and Kirby’s method was confirmed as a standard method for susceptibility testing. This method has potential for the routine testing of susceptibility in clinical laboratories. Furthermore, the method is widely accepted because it offers a simple, cost-effective protocol for the detection of multiple targets [24]. However, along with these advantages, it also has some significant drawbacks: only semi-automation is available (Sirscan), insufficient data availability for many bacteria (strains of *Pseudomonas*, *Bacillus*, and *Corynebacterium*), and it has a poor performance when analyzing slow-growing and fastidious bacteria [25,26]. Influenced by many physiochemical factors like evaporation, solubility, pH, temperature, and nutrient media, additional limitations restrict its suitability for accurate diagnostics [27]. 

Recently, the emergence of various instruments for analyzing the zone of inhibition has added to the reliability of the disk diffusion results by reducing variability due to operator handling and interpretation. The camera or scanner takes the picture, and the inbuilt image analysis software displays the zone of inhibition and compares the obtained results with the various guidelines present in the database. Accuzone (AccuMed International, Hillsboro, Oregon, USA), Biomic (Giles Scientific, Santa Barbara, California, USA), Mastascan Elite (Mast, Bootle, UK), and Sirscan (Becton Dickinson, Oxford, UK) are a few of the instruments capable of analyzing the zone of inhibition, but all differ in data input, analysis, ease of use, and presentation of results [28].

### 2.2. Dilution

Dilution was one of the earliest tools in microbiological practice, starting in the early 1870s, and it allows the growth and identification of bacterial populations in suspension [31]. Pasteur, Lister, Koch, and Ehrlich were listed as the pioneers in the field of bacteriology, and they worked on the concept of macrodilution [32]. William Roberts and John Tyndall further contributed to the macrodilution method and observed bacterial growth in a diluted medium [33]. The two basic types of dilution are microdilution and macrodilution, wherein broth and agar are the most commonly used mediums. In broth dilution, consecutive two-fold dilutions (1, 2, 4, 8, and 12 µL) of antibiotics are made and dispensed into micro-centrifuge tubes containing bacterial growth medium, followed by making up the final volume by adding the medium and incubating overnight at 35 °C. Finally, the growth examination is carried out for setting the breakpoint through the turbidity of culture media (Figure 2) [34,35]. In agar dilution, antibiotics are diluted into the agar medium, followed by plate formation and application of bacterial cells to the surface of the agar plate.

In the early 20th century, various scientists made efforts to introduce serial dilution. They set the dilution factor in terms of geometric progression, and derived the generalized mathematical equation for interpreting the dilution results [36,37,38,39,40]. In 1929, Alexander Fleming performed the serial dilution technique to understand the activity of antibiotics. In this technique, two-fold dilution of antibiotics is mixed with a pre-inoculated liquid medium to determine antibiotic actions by checking the turbidity. In 1942, Fleming modified the previous protocol by using pH instead of turbidity to identify antibacterial activity. In the same year, Rammelkamp and Maxon introduced broth macro dilution, or the “tube dilution method”, which is regarded as the standardized dilution method for both minimum inhibitory concentration (MIC) and AST. CLSI recommends guidelines to set the breakpoints. The first attempt regarding AST was made by Schmith and Reymann using agar medium during the 1940s [41]. 

Microdilution is a miniaturized prototype of the macrodilution method where susceptibility testing is performed on disposable 96-well microtiter plates, where each well has a sample capacity of ~0.1 mL (Figure 2) [42]. To dispense the samples into microwells, mechanized dispensers are used to avoid the handling error. After overnight incubation, growth and MIC are assessed through specialized optical instruments. This method has been well standardized for most fastidious bacteria [13]. 

The central drawback of dilution methods is the requirement of a large volume of reagents. Apart from that, other potential limitations include: experimental space, tedious dilution steps (macrodilution), the possibility of false positive results due to long incubation times [43], chances of cross-contamination, bacterial incompatibility for growth, and the inability of discriminating viable and nonviable bacteria. Maintaining the recommended optimum testing parameters like pH, temperature, media, and length of incubation are additional hurdles, and a control viability plate is mandatory in tests to achieve practical clinical relevance [44]. 

### 2.3. Etest

Epsilometer testing (Etest) is another significant development for the routine analysis of widespread antibiotic resistance in bacteria. In the late 1980s, Bolmström and Eriksson developed this test [45]. AB BIODISK manufactured the first Etest plastic strip to inspect multiple antibiotics on a single platform in 1991 (Figure 2) [45]. Etest plastic strips are coated with pre-defined antibiotic concentrations, and the corresponding interpretive MIC ranges are marked on the surface and back of the strip, respectively. For detection, multiple strips are placed on a pre-inoculated streaked agar plate, followed by an overnight incubation; elliptical inhibition zones appear around the strips, indicating the MIC at the intersection point between the inhibition zone and the strip edge [46]. The simplicity, accuracy, and reliability of the Etest makes it appropriate and convenient for Food and Drug Administration (FDA) approved commercialization [47]. The ability of convenient interpretations of MIC under diverse physical conditions made the Etest a preferential method over standardized disk diffusion and dilution techniques in clinical laboratories for AST [30,48].

In the 1990s, series of comparative studies with the other standardized techniques, for instance, agar dilution and diffusion, and broth dilution, established the significance of the Etest. Many strains of *H. pylori*, *Neisseria gonorrhoeae, Enterococcus* spp., and many other clinical isolates were tested by Etest and compared with standard methods, resulting in a good correlation in the range of 91%–99% [49,50,51]. Recently, in 2016, methicillin-sensitive *S. aureus* (MSSA) and methicillin-resistant *S. aureus* (MRSA) isolates were examined with an Etest to determine the MIC of ceftaroline. The results were compared with broth microdilution (BMD) and showed an excellent agreement of more than 95% [52,53]. Multiple cultures of *Campylobacter* spp. against seven antibiotics were also evaluated by Etest to determine their resistance [54]. All these results demonstrated the reliability and importance of the Etest in evaluating MICs of a wide range of antibiotics over the present standardized methods, especially for slow-growing bacteria (*Campylobacter jejuni*, *H. pylori*) and rare fastidious bacteria (*S. pneumoniae* and *Neisseria spp.*). One of the significant advantages of the Etest is its sensitivity; it can detect extended-spectrum beta-lactamase (ESBL), even in a trace amount [55]. Additionally, accurate resistance strains can easily be easily quantified in laboratories/hospitals due to the stable concentration gradient of antibiotics marked on the Etest strip.

Besides several advantages, there are some limitations that cannot be ignored, primarily related to the inaccurate and inconsistent behavior of the Etest for certain antibacterial agents, such as Penicillin, ciprofloxacin, ofloxacin, and rifampicin [56]. Some additional demerits that make the Etest complicated for routine test analyses are: pH-sensitive coated antibiotics, expensive batch performance, strip storage, and laboratory set-up for proper plate inoculation and incubation [57].

### 2.4. Matrix-Assisted Laser Desorption Ionization-Time of Flight Mass Spectrometry (MALDI-TOF MS)

MALDI-TOF MS, introduced in 2000, is another sensitive method for bacterial identification. High sensitivity and accuracy are the key characteristics that make it a useful method for clinical relevance. Assorted studies reveal its significance in discriminating MRSA, MSSA, and other bacterial strains where susceptible and resistant bacteria have been evaluated through spectral peak analysis. Even the subtle difference in expression profiles have been noticed in isogenic strains of *S. aureus* [58,59]. The efficiency of MALDI-TOF MS has been further investigated on vancomycin-resistant Enterococci, where sensitivity higher than 90% has been recorded. Furthermore, analysis of multiple targets with different resistant strains of *Pseudomonas spp.* against ciprofloxacin, tobramycin, and meropenem have been identified efficiently [60]. The newly developed MALDI Biotyper antibiotic susceptibility test rapid assay (MBT-ASTRA) is a more-straightforward and cost-effective modulation of MALDI-TOF MS used for both AST and MIC determination [61]. Despite all the advantage of MALDI-TOF MS, the expensive nature of the instrument and its maintenance are prime disadvantages for mass application.

### 2.5. Automated Systems

Since the dawn of automated technologies in the 1980s, antibiotic susceptibility tests have been perpetually improvised and, hence, have superseded conventional phenotypic methods [13]. Automation, simplicity, and compactness are the major reasons for their widespread acceptance in diagnostics. Computer integration has allowed online analysis and data sharing, which is a giant leap for results validation, especially in remote areas [62]. Among the developed automated systems, MicroScan WalkAway (Beckman Coulter, Inc. Atlanta, Georgia, USA) (1980), Micronaut (Merlin, berlin, Germany) (1990), the avantage test (Abbott Laboratories, Irving, Texas, USA) (1980), Vitek 2 (bioMe’rieux, Marcy-l’Étoile, France) (2000), Phoenix (BD Diagnostics, Franklin Lakes, New jersey, USA) (2001), and Sensititre ARIS 2X (Trek Diagnostic Systems, Oakwood Village, Ohio, USA) (2004) are the major FDA approved systems for AST. Vitek and Pheonix detect growing bacteria on the basis of turbidity, whereas comparable automated systems like MicroScan WalkAway (Beckman Coulter, Inc. Atlanta, Georgia, USA) and Sensititre ARIS 2X (are based on fluorescence emission of the growing bacteria. The resistance in gram-negative, gram-positive, and *Streptococcus* strains of bacteria can easily be estimated through Phoenix, and Vitek 2, but, MicroScan WalkAway and Sensititre ARIS 2XESBL (Trek Diagnostic Systems, Oakwood Village, Ohio, USA) are capable of detecting the extended-spectrum beta-lactamase (ESBL)-producing strains in the species mentioned above [63]. Micronaut and Avantage are capable of accurate direct susceptibility testing for gram-positive and gram-negative bacteria, respectively [64,65].

Detection incompatibility for many bacteria/antibiotics using Sensititre ARIS 2 and Vitek 1 have led to the development of the updated Sensititre ARIS 2X and Vitek 2, which have better performances and are applicable to a broader range of bacteria/antibiotics. Presently, all the automated systems are incorporated with advanced expert system software for enhanced performance and online data processing [28]. Every automated system has a specific panel capacity and an average time for performing detection, which varies from 40 to 100 wells, and can vary in time such as 20, 12 and 9 h, respectively. Certain models such as Phoenix AP, and Vitek 2Xl, are dedicated towards automated inoculation, enhanced card capacity and compactness.

The aforementioned systems lack reproducibility, sensitivity, and reliability compared with the existing traditional methods. Moreover, an inability to test a wide range of clinically relevant bacteria (e.g., *S. pneumonia*), antimicrobial agents (e.g., vancomycin), and heteroresistant isolates, as well as a limited panel capacity and the high cost of instruments and consumables, are all significant issues that restrict these systems from frequent analysis [66].

## 3. Genotypic AST Methods

Molecular or genotypic AST are the effective direct methods that eliminate tedious bacterial cultures, long incubation, chances of contamination, and the spreading of deadly infections [67]. PCR, DNA microarray and DNA chips, and loop-mediated isothermal amplification (LAMP) are some of the genotypic techniques for the detection of antibiotic resistance. Mutational assessment of methicillin resistance in *Staphylococcus spp*., vancomycin resistance in *Enterococcus spp*., and multi-antibiotic (isoniazid, rifampin, streptomycin, pyrazinamide, and the fluoroquinolones) resistance in *Mycobacterium* spp. have been successfully estimated through various genotypic techniques. 

**Figure 3:**
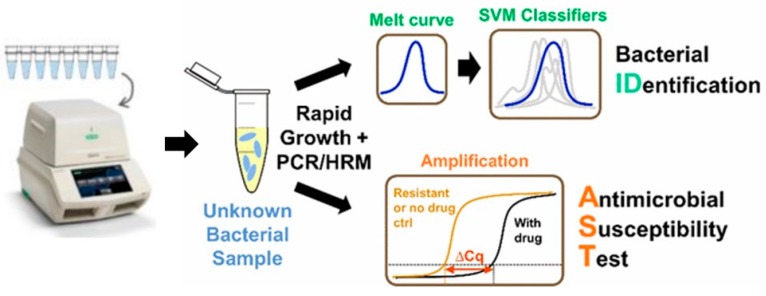
Digital PCR-High Resolution Melt analysis (HRM)-based bacterial identification from mixed bacterial samples, reproduced with permission from [71], published by American Chemical Society, 2017. (SVM: Support-vector machine)

PCR is one of the most efficient and rapid molecular tools for quantification and profiling of bacterially infectious genes. The first report on PCR diagnostic application was published by Saiki et al. [68]. The general methodology of PCR includes cycles of denaturation, annealing of the primers, and elongation of the primers by a thermostable DNA polymerase in a compatible buffer containing nucleotides, ions, and so on. Each cycle of amplification doubles the target DNA molecule. The amplified target can be confirmed for the presence of resistance genes through electrophoresis, southern blotting, restriction fragment-length polymorphism, single-strand conformation polymorphism (SSCP), DNA fingerprinting, molecular beacons, and other DNA sequencing analysis methods (Figure 3) [68,69]. Another tool developed on the basis of PCR is LAMP, which has also been used for the evaluation of AST. In LAMP, the gene of interest is amplified at a constant temperature of 60–65 °C using a *Bst* DNA polymerase instead of *Taq* polymerase because of strong strand displacement activity (required in isothermal techniques) [70]. 

DNA microarrays and DNA chips are the other promising technologies utilized for screening susceptibility [72]. DNA arrays employ cDNA fragment probes on nylon membrane, where each DNA chip has a glass or silicon platform for probe binding. The specific hybridization of the labeled probe with the target and its recognition help to determine the resistance. Determination of isoniazid resistance in *M. tuberculosis* has been carried out successfully through DNA microarrays and chips [73,74]. Colorimetric detection and multiplexing are the attractive features of these techniques.

Genotypic methods are generally attributed to the rapid, direct, sensitive, and specific detection of resistance genes, but they also suffer from severe drawbacks that diminish their clinical utility. These drawbacks include: (i) the individual antimicrobial agents to be tested need a specific assay for detection; (ii) only potential/key resistance genes can be detected, which are often not relevant due to coincidental mutations; (iii) there is a lack of sensitivity towards the patients with latent infections, or when only a few organisms are present in a sample; (iv) the genetic mechanism/profile for the resistance of all bacteria is not yet defined; (v) the occurrence of false-positive results due to contamination of the test sample might be expected; (vi) they require expensive reagents and machinery with specific maintenance conditions; and most importantly (vii) all the tools have a prerequisite of skilled personnel [67].

## 4. Emerging Methods for AST

Microfluidics-based diagnostics are one of the most promising emerging tools for AST. Microfluidics is an evolving field characterized by the manipulation of fluids in micro-volume, thereby offering portability, cost-effectiveness, multiplexing, reproducibility, and a controllable environment in an in vitro system [12]. The concept was first introduced in the semiconductor and micro-electromechanical systems (MEMS) industries, then further extended to the field of biomedical research [75]. Integrated microfluidic devices, generally referred to as micro total analysis systems (µTAS), are proficient in performing molecular diagnostics [76].

The quantity of samples has always been the biggest challenge for biological studies in general, and pathological analysis in particular. Since microfluidics is capable of dealing with the minimal quantity of samples, it has therefore emerged as a promising tool for pathologists. Currently, microfluidics platforms are capable of single-cell analysis, and can even analyze the single-cell interrogation of signaling networks in cultured cell lines. In the past decades, numerous conventional and automated strategies have been successfully embraced to diagnose the ever-increasing antibiotic resistance in bacteria [77]. Nevertheless, the most-practiced techniques, such as disk diffusion and microdilution, have a high chance of cross-contamination, laborious protocols, lengthy processing times, improper power supplies, and cumbersome set-ups that render their application in resource-limited regions, and unfortunately, these regions have a higher rate of resistance too [2,6]. The rise of microfluidics as a diagnostic tool has shown promise in addressing the abovementioned shortcomings [78,79].

Progression in optical imaging has developed various image sensors with high sensitivity and resolution that are capable of biological analysis. These imaging systems are frequently used for morphological and growth studies of bacteria. Generally, owing to real-time analysis and minimum culture dependency, microfluidic devices coupled with an optical sensor can perform AST and detect the MIC in few hours [80]. Recent reports based on single bacterial cell analysis have claimed that optical sensor-based nanofluidic (30 nl) can finish AST within 30 min [81]. This direct imaging of single bacterium requires simple sample preparation steps, but eliminates the tedious steps of continuous sample injection, loading of cells, and counting-based cell identification [81].

**Figure 4:**
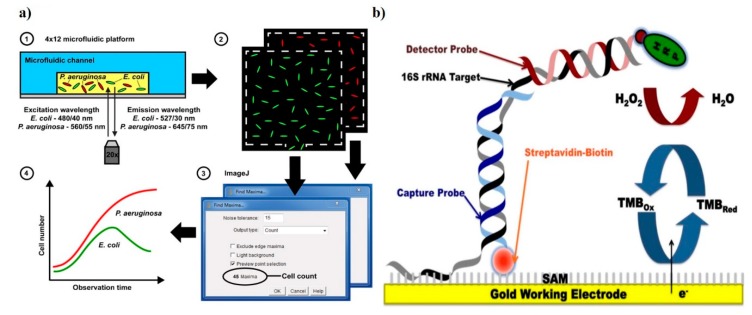
Demonstration of sensors for antibacterial susceptibility testing involving (**a**) an optical microfluidics biosensor, showing optical detection of microbial cultures, reproduced with permission from [83], published by RCS Advances, 2015; and (**b**) an electrochemical biosensor, detection was based on hybridization of the target bacterial 16S rRNA with a detector probe, adapted with permission from Liu [86] (under terms of the Creative Commons attribution license).

Fluorescence proteins and dyes are commonly used for tagging resistant biomarkers. These proteins/dyes can be of biological or chemical origin. One of the pioneer proteins used for imaging is green fluorescent protein (GFP). GFP, obtained from jellyfish *Aequoria victoria,* is essential for the noninvasive real-time monitoring of antimicrobial susceptibility [82]. Adding to this, the same group was able to evaluate multiple antibiotic sensitivities in real-time in polymicrobial culture bacterial strains (namely, *E. coli*, *P. aeruginosa,* and *K. pneumoniae*) simultaneously. Green and red fluorescent protein tagging have been utilized for real-time growth quantification in the presence of multiple antibiotics on a multiplexed microfluidic platform (Figure 4) [83]. All these methods are sensitive and reliable, but creating these recombinant bacteria requires molecular handling, which is a challenge for routine clinical observations. Fluorescent dyes are another means for optical fluorescence tagging. These chemicals, with all the benefits of the fluorescence protein, can avoid stearic hindrance due to their small size, and elimination of cloning or transformation. SYTOX green and resazurin (alamarBlue; inactive precursor of resazurin) are two common fluorescence indicator dyes used for viability testing in AST [84,85]. Similarly, resazurin can be utilized in colorimetric detection. When the culture media is supplemented with antibiotic and resazurin, a uniform intense blue color is obtained in the beginning. In the presence of resistance, the resazurin is reduced to resafurin, and the intense blue color changes to pink and leuco; in the absence of resistance in bacteria, the blue color sustains. This method is also translated to a microfluidic chip for MIC estimation for four different antibiotics against 20 clinical strains of *Escherichia* and *Shigella* [85].

Similarly, bioluminescence or ATP bioluminescence assay (ATP-BLA) is an enzyme-based approach mediated by luciferase enzyme that converts luciferin substrate to oxyluciferin in the presence of ATP, leading to an emission of light [87]. By using this phenomenon, the susceptibility of 13 different types of clinical strains present in urinary infections were evaluated against eight antibiotics on the microfluidic plate. When bacteria grew in the presence of antibiotics, resistant bacteria resulted in bioluminescence, whereas susceptible bacteria remained neutral. Both identification and susceptibility were obtained within 3–6 h [88,89]. 

Several electrochemical devices have been developed for AST. One of the most prominent works utilized AC electrokinetic fluid motion and Joule heating-induced temperature elevation for the electrochemical sensing of bacterial 16S rRNA (Figure 4) [86]. Real-time and rapid detection are possible, as 16s rRNA is highly specific for the bacterial pathogens in blood culture and does not need prior purification. Although 16S rRNA has indeed provided critical details about sensitive bacterial analysis, genetic complexities across kingdoms make it inappropriate for reproducible, clinically relevant, or point-of-care susceptibility testing. The use of electroactive chemicals (redox reagents) as probe molecules can be an interesting approach to elucidating bacterial susceptibility. In 2015, pyocyanin, a potential marker of cell viability and virulence, was studied for the electrochemical monitoring of the susceptibility of *P. aeruginosa* biofilms on a microfluidic device [90]. A good correlation between the electrical signal drop and the viability of *P. aeruginosa* cells in the presence of antibiotics was successfully demonstrated. The most recent electrochemical biosensor can perform AST within 90 min along with the label-free isolation of bacteria from whole blood samples [91]. Plastic-based microchips with printed electrodes capture the target bacteria with the help of specific antibodies. The electrochemical response to the captured bacteria is monitored in both the presence and absence of antibiotics.

The simplified blood culture system (SBCS) is an emerging tool in near-patient testing and surveillance tools for blood stream infections (BSI) [92]. SBCSs uses unprocessed samples, thereby, it requires zero sample preparation time. Furthermore, SBCSs combine detection, gram status, and identification in blood culture (BC) instruments, thus ensuring the evaluation of susceptibility within 8–12 h, whereas the conventional blood culture system requires up to 48 h because of incubation for colony generation and susceptibility testing. Moreover, owing to its simplified and time efficient nature, the SBCS can be used in resource-limited settings, which was not possible for conventional BC methods due to the lack of electricity, limited culture bottles, profuse dust, improper ambient temperature control, and lack of skilled personnel [92]. 

## 5. Challenges and Future Perspective

Primarily, the continuous flow of culturing media to feed cells is the biggest challenge for maintaining nutrient conditions, as a slight change in growth media due to evaporation can affect the bacterial growth and hence the accuracy of AST [93,94]. The accuracy of AST might be improved by considering the factors altering its pharmacokinetics (diffusion, metabolism, and elimination), which may result in unpredictable changes, and therefore, the MIC might change. Furthermore, to avoid the indiscriminate use of antibiotics and the evolution of antibiotic resistance, the dosage scheme should also consider the method of antibiotic administration (e.g., oral versus intravenous (IV) administration) and the site of infection, along with the AST results [95]. 

The applicability of molecular biology tools such as cloning and recombinant expression has enhanced the sensitivity of detection, and low concentrations of bacteria in clinical samples can be evaluated, but there are some serious limitations. Creating recombinant strains with these molecular genes is a troublesome process. Firstly, genetic analysis is cumbersome and prone to mutations due to frequent change in the resistant behavior of bacteria [96]. Therefore, prior knowledge of specific resistance genes before susceptibility testing is essential. Secondly, the genetic markers for all clinically relevant bacteria is still unknown, moreover, the known targets are not universal. Thirdly, advanced molecular biology skills and laboratory sets are important in dealing with recombinant technology. Alternatively, fluorescence label dyes are used to avoid molecular challenges. Although the use of dyes is simple and easy over other molecular techniques, the requirement of a high-resolution A charge-coupled device camera (CCD) and sophisticated instruments for signal amplification and observation are restricted to resource-limited areas. False-positive results due to changes in physical parameters are also a major problem. Additionally, immense versatility among the culture conditions of different bacterial species is a challenge for the development of a single platform for different bacteria and multiplexing [97,98].

Looking beyond the imaging requirements, researchers must also focus on other relevant unanswered questions in the context of diagnostic devices. This primarily includes what performance metrics will be essential to lessen the exposure of contamination from hospitals or biomedical research units, and secondarily, what advances will be necessary to reduce the incorporation of sophisticated external circuits, pumps, and pneumatic systems in maintaining the flow continuity in microfluidic platform To address these issues, the paper-based detection system seems to be an attractive approach in developing a cost-effective, automated, and incinerable platform for the determination of susceptibility. In the past decades, the emergence of paper-based microfluidics has proven its performance for biological assays [99], and it could be a bedrock for reducing contamination through easy disposal. Additionally, the inbuilt property of capillary action of paper can eliminate the integration of complicated pneumatic chambers and pumps. Therefore, more research work on paper microfluidic AST would be beneficial in the future. An absence of the simultaneous detection of multiple analytes and the need for simpler fabrication tools are further shortcomings of microfluidics. The collaboration of academic research and commercial firms with transparent technology distribution might offer a compelling solution for multiplexing and for more-straightforward fabrication. Hence, efforts to expand our understanding, especially for the development of a user-friendly device with multiplexing, would be valuable.

Smartphones and the technology that powers them are continually growing more advanced. Coupling fluorescent/colorimetric tools with the ubiquitous and ever-evolving smartphones will enable us with on-site monitoring, real-time database updates, and the generation of an antibiotic susceptibility map to help us understand the geographical prevalence of resistance. The use of smartphones is limited by their camera performances, which result in lower detection limits, especially in a colorimetric assay [100]. It is exciting to speculate that ongoing advancement will bring much higher resolution cameras coupled with better time-lapse technologies and offer morphological and biochemical measurements. 

Loop-mediated isothermal amplification polymerase chain reaction (LAMP-PCR) has shown us the way to develop lateral-flow devices for the genetic detection of antibiotic resistance. Carbapenem-resistance in *Acinetobacter baumanii* was successfully evaluated by LAMP-PCR by amplifying the OXA-type carbapenemases and metallo-β-lactamases genes [101]. However, more studies are warranted to establish the applicability of LAMP. In coming years, there might be a rise in non-infecting but resistance-bearing mutants; genetic detection will be essential to screen out these mutants, and LAMP-based lateral flow devices will serve that purpose.

## 6. Conclusions

While all AST methods offer qualitative assessments using susceptible, intermediate, or resistant categories, certain methods specify qualitative and effective antibiotic dosage (e.g., minimum inhibitory concentration) and formulate a profile of empirical therapy for the proper management of individual patients’ health against deadly infections. A rise in antibiotic resistance is a certainty, therefore we must develop technologies that will permit rapid AST (within an hour) and are non-invasive (saliva- or urine-based) or minimally invasive. 

## Supplementary Materials

The following are available online at https://www.mdpi.com/2075-4418/9/2/49/s1.
