# Supplementary material for: Current and Emerging Methods of Antibiotic Susceptibility Testing
Source: Diagnostics (Basel). 2019 May 3;9(2):49. doi: 10.3390/diagnostics9020049 (PMC6627445; doi:10.3390/diagnostics9020049)
Supplement: Supplementary file 1 [file diagnostics-09-00049-s001.pdf]

Supplementary Table 1. Major antibiotic resistant bacteria, clinical manifestation, associated and available antibiotics for treatment (only examples are given).

**Resistant Bacteria/Type/Region of High Prevalence**	**Symptoms/Representative Clinical Infections**	**Antibiotics Associated with Resistance**	**Available Treatments**	**Ref**
Staphylococcus aureus (S. aureus) **(MRSA)/ gram-positive, cocci, Eastern Mediterranean Region (0-92%)**	Skin and soft tissue infections, toxic shock syndrome, osteomyelitis pneumonia/lung infection, endocarditis, meningitis, urinary tract infection (UTI)	beta-lactam antibiotics (e.g., penicillin, oxacillin, monobactams, nafcillin, amoxicillin, and most cephalosporins) erythromycin	Tedizolid, tigecycline, vancomycin or daptomycin, ceftaroline, telavancin; linezolid; dalbavancin, oritavancin, clindamycin, minocycline, doxycycline, linezolid	[1-5]
S. aureus **(VISA/hVISA/VRSA)/ gram-positive, cocci, South-East Asia Region (3.42–6.81%)**	Skin and soft tissue infections, UTI, bacteraemia, pneumonia/ lung infection, osteomyelitis, endocarditis, meningitis	beta-lactam antibiotics, vancomycin, erythromycin	Minocycline, or quinupristin-dalfopristin, daptomycin, linezolid, telavancin, ceftaroline	[2,6-9]
Streptococcus pyogenes **/ gram positive, cocci, Europe region (4–40%)**	Pharyngitis, necrotizing fasciitis, toxic shock syndrome, autoimmune diseases	macrolides, clindamycin, and lincosamide	Penicillin, cephalosporins, macrolides, and clindamycin	[10-14]
Streptococcus pneumoniae **/ gram-positive, diplococcus, African Region (1-100%)**	Pneumonia/ lung infection, otitis media, sinusitis, bronchitis, bacteraemia, peritonitis, cellulitis, arthritis, meningitis	optochin, ceftriaxone, cefotaxime, penicillin, Cefuroxime, cephalosporins, erythromycin, tetracycline, doxycycline	Vancomycin +/- rifampin; gemifloxacin, moxifloxacin, levofloxacin) linezolid; clindamycin; imipenem/cilastatin, ceftriaxone plus	[2,5,15-17]
Escherichia coli **- CTX-M extended spectrum beta-lactamases (ESBL)/ gram- negative, rod, South-East Asia Region 19-95% ()**	UTI, diarrhoea, colitis	Penicillin, cephalosporins, fluoroquinolones, aztreonam	Meropenem, carbapenems (ertapenem, doripenem), imipenem, cilastatin, fosfomycin, trimethoprim-sulfamethoxazole, cefepime	[5,18-22]
Enterococcus faecium **(VRE)/ gram- positive, oval cocci Latin America (0-48%)**	Pelvic infections, endocarditis, neonatal infections and UTI	Vancomycin, gentamicin, ampicillin, beta lactams and aminoglycosides, streptomycin	Linezolid, daptomycin, streptogramins tigecycline, quinupristin-dalfopristin, sultamicillin	[23-30]
Pseudomonas aeruginosa **(MDR)/gram-negative, rod Latin America (0-48%)**	Skin and soft-tissue infections, UTIs, bloodstream infections, pneumonia/lung infection, endocarditis, meningitis	Imipenem/cila-statin, mero-penem, non-antipseudo-monal penicillins, cephalosporins	Colistin, ceftazidime-avibactam or ceftolozane-tazobactam, polymyxin B	[31-37]
Klebsiella pneumoniae **(ESBL)/ gram- negative, rod South-East Asia Region (20-72%)**	Bacteraemia, sepsis, pneumonia, UTIs, conjunctivitis, and surgical site infections	Beta-lactam, ciprofloxacin, cephalosporins; aztreonam; aminoglycosides and norfloxacin	Carbapenems, doripenem, flomoxef, Piperacillin ceftolozane-tazobactam, ceftazidime-avibactam	[5,38-42]

Mycobacterium tuberculosis **(MDR-TB)/ acid-fast Indo-Pak Region (1.6-21.6%)**	Tuberculosis (lung infection), osseous tuberculosis, night sweats, fever, weight loss	Isoniazid; rifampin; streptomycin	Pyrazinamide, a fluoroquinolone, an injectable agent (i.e., amikacin), ethionamide, cyclomerize, linezolid, clofazimine	[43-48]
Acinetobacter baumannii **/ gram-negative, coccobacillus South-East Asia Region (25-95%)**	Pneumonia, UTI, septicaemia, Meningitis, Wound and surgical site infections	Beta-lactams, imipenem, meropenem, antipseudomonal agents, and fluoroquinolones,	Polymyxins, Polymyxins + carbapenem, minocycline, tigecycline, or rifampin,	[49-53]
S. epidermidis **/gram- positive, cocci Africa (0-34%)**	Bacteraemia and sepsis, catheter, implant, endocarditis and prostheses-related infection	Methicillin, penicillin, amoxicillin rifamycin, flouroquinolones, chloramphenicol, erythromycin, clindamycin, and sulfonamides	Vancomycin, vancomycin +/- (rifampin + gentamicin) daptomycin, linezolid, quinupristin-dalfopristin	[54-57]
Burkholderia cepacia **/ gram-negative, rod USA (0-40%)**	Necrotizing pneumonia, peritonitis, liver abscesses	Chloramphenicol, Polymyxins, ciprofloxacin, moxifloxacin, colomycin, and aminoglycoside	Clinafloxacin, meropenem, ceftazidime and high-dose tobramycin (multiple combination is preferred)	[58-64]
Neisseria gonorrhoeae **/ gram-negative, diplococci European Region**	Pelvic inflammatory disease, chronic pelvic pain, ectopic pregnancy, neonatal conjunctivitis, and infertility	Penicillin, ceftriaxone	Ciprofloxacin, spectinomycin and third-generation cephalosporins such as ceftriaxone and cefixime	[5,65,66]
Clostridioides difficile **/ gram-positive, rod shaped North America (0-10%)**	Diarrhoea, colitis	Cefotaxime, levofloxacin, erythromycin, Cephalosporins	Rifampicin, meropenem, rifaximin	[67-70]

Supplementary Table 2. Principal AST methods, associated advantages and disadvantages (only examples are given).

Methods	Technique	Major advantages	Major disadvantages	References
**Phenotypic**	Disk diffusion	Simple, cost effective, flexible & well standardized	Lacks qualitative test & full- automation, risk of contamination, time consuming (minimum of 10 hours), no MIC determination, needs at least cells (~10^5)	[71]
	Microdilution	Miniaturized, time feasible, allows MIC determination with multiple sampling	Inflexibility of drug selections available in standard commercial panels, time consuming (minimum of 10 hours), needs at least cells (~10^5)	[72-75]
	Macrodilution	Simple, cost effective, allows MIC determination	Tedious sample preparation, risk of contamination, time consuming (minimum of 10 hours), needs at least cells (~10^5) and expensive technique.	[76,77]
	Epsilometer test	Detection of fastedious bacteria, easy to perform	High rate of false positive results, proper storage of Etest strip, (minimum of 10 hours), needs at least cells (~10^5)	[78-82]
	MALDI-TOF MS	High throughput quick sample preparation automation possible	Expensive (), needs at least cells (~10^5) expensive, different genetic markers	[83-85]
**Genotypic**	PCR	Quick (2-4 hours), simple and cost-effective, highly reproducible, high specificity, low sample requirement, Multiplexing,	Unknown parameters (mutation, G and C content, porosity of gel matrix, ionic strength and pH) can affect analysis, false positive results, expertise is required for analyses	[86,87]

	DNA microarrays/chips	high sensitivity (picogram of nucleic acid) Multiplex detection, automated specific and high sensitivity (picogram of nucleic acid)	Labor intensive procedure to synthesize, purify, and store DNA before microarray fabrication, problem of cross hybridization	[88-90]
**Automated**	Vitek	Easy and fast data management, reproducible	Narrow antibiotic range requires more cells (~10^5)	[91,92]
	Sensititre	Automated, rapid and simple operation	Narrow antibiotic range, limited panel capacity, expensive, needs at least cells (~10^5)	[93-95]
	MicroScan Walkaway	Validate susceptibility with expert software system, accurate & data sharing	long incubation (minimum of 12 hours), expensive, needs at least cells (~10^5)	[96-98]
	Micronaut	Direct susceptibility test	High false positive, needs at least cells (~10^5)	[99]
	AvantagEtest	Effortless, direct test	Discrepancies in results with some antibiotic combinations	[100-102]
**Emerging AST**	Optical	Real-time quantification of bacterial growth dynamics, time efficient (1-4 hours), morphological analysis, single cell detection	Requires microscope, cloning, genetic modifications may affect the growth dynamics	[103-105]
	Electrochemical	High sensitivity (10^3 cells), economical, time efficient time efficient (1-4 hours)	Narrow temperature range Short life time of sensors interference with electroactive agents of sample	[106-108]

References

1. Reygaert, W. Methicillin-resistant Staphylococcus aureus (MRSA): identification and susceptibility testing techniques. *Clin Lab Sci* **2009**, *22*, 120-124.
2. Tong, S.Y.C.; Davis, J.S.; Eichenberger, E.; Holland, T.L.; Fowler, V.G. Staphylococcus aureus Infections: Epidemiology, Pathophysiology, Clinical Manifestations, and Management. *Clinical Microbiology Reviews* **2015**, *28*, 603-661, doi:10.1128/CMR.00134-14.
3. Guignard, B.; Entenza, J.M.; Moreillon, P. Beta-lactams against methicillin-resistant Staphylococcus aureus. *Curr Opin Pharmacol* **2005**, *5*, 479-489, doi:10.1016/j.coph.2005.06.002.
4. Scordo, K.A. Intravenous Antibiotics Used in the Treatment of Methicillin-Resistant Staphylococcus Aureus. *AACN Adv Crit Care* **2015**, *26*, 233-243, doi:10.1097/nci.0000000000000095.
5. Organization, W.H. *Antimicrobial resistance: global report on surveillance*; World Health Organization: 2014.
6. Entenza, J.M.; Bétrisey, B.; Manuel, O.; Giddey, M.; Sakwinska, O.; Laurent, F.; Bizzini, A. Rapid Detection of Staphylococcus aureus Strains with Reduced Susceptibility to Vancomycin by Isothermal Microcalorimetry. *Journal of Clinical Microbiology* **2014**, *52*, 180-186, doi:10.1128/JCM.01820-13.
7. Liu, C.; Chambers, H.F. Staphylococcus aureus with Heterogeneous Resistance to Vancomycin: Epidemiology, Clinical Significance, and Critical Assessment of Diagnostic Methods. *Antimicrobial Agents and Chemotherapy* **2003**, *47*, 3040-3045, doi:10.1128/AAC.47.10.3040-3045.2003.
8. Hiramatsu, K.; Kayayama, Y.; Matsuo, M.; Aiba, Y.; Saito, M.; Hishinuma, T.; Iwamoto, A. Vancomycin-intermediate resistance in Staphylococcus aureus. *Journal of Global Antimicrobial Resistance* **2014**, *2*, 213-224, doi:https://doi.org/10.1016/j.jgar.2014.04.006.
9. Zhang, S.; Sun, X.; Chang, W.; Dai, Y.; Ma, X. Systematic Review and Meta-Analysis of the Epidemiology of Vancomycin-Intermediate and Heterogeneous Vancomycin-Intermediate Staphylococcus aureus Isolates. *PLoS One* **2015**, *10*, e0136082-e0136082, doi:10.1371/journal.pone.0136082.

10. Broaddus, V.C.; Light, R.W. Pleural effusion. In *Murray and Nadel's Textbook of Respiratory Medicine (Sixth Edition)*, Elsevier: 2016; pp. 1396-1424. e1310.
11. Murray, P.R.; Wold, A.D.; Schreck, C.A.; Washington Ja, I.I. Effects of selective media and atmosphere of incubation on the isolation of group A streptococci. *Journal of Clinical Microbiology* **1976**, *4*, 54-56.
12. Shulman, S.T.; Bisno, A.L.; Clegg, H.W.; Gerber, M.A.; Kaplan, E.L.; Lee, G.; Martin, J.M.; Van Beneden, C. Clinical practice guideline for the diagnosis and management of group A streptococcal pharyngitis: 2012 update by the Infectious Diseases Society of America. *Clin Infect Dis* **2012**, *55*, 1279-1282, doi:10.1093/cid/cis847.
13. Walker, M.J.; Barnett, T.C.; McArthur, J.D.; Cole, J.N.; Gillen, C.M.; Henningham, A.; Sriprakash, K.S.; Sanderson-Smith, M.L.; Nizet, V. Disease Manifestations and Pathogenic Mechanisms of Group A Streptococcus. *Clinical Microbiology Reviews* **2014**, *27*, 264-301, doi:10.1128/CMR.00101-13.
14. Efstratiou, A.; Lamagni, T. Epidemiology of Streptococcus pyogenes. In *Streptococcus pyogenes: basic biology to clinical manifestations [Internet]*, University of Oklahoma Health Sciences Center: 2017.
15. Blaschke, A.J. Interpreting Assays for the Detection of Streptococcus pneumoniae. *Clinical Infectious Diseases: An Official Publication of the Infectious Diseases Society of America* **2011**, *52*, S331-S337, doi:10.1093/cid/cir048.
16. Pikis, A.; Campos, J.M.; Rodriguez, W.J.; Keith, J.M. Optochin Resistance in Streptococcus pneumoniae: Mechanism, Significance, and Clinical Implications. *The Journal of Infectious Diseases* **2001**, *184*, 582-590, doi:10.1086/322803.
17. Kaplan, S.L.; Mason, E.O., Jr. Management of infections due to antibiotic-resistant Streptococcus pneumoniae. *Clin Microbiol Rev* **1998**, *11*, 628-644.
18. Molina, F.; López-Acedo, E.; Tabla, R.; Roa, I.; Gómez, A.; Rebollo, J.E. Improved detection of Escherichia coli and coliform bacteria by multiplex PCR. *BMC Biotechnology* **2015**, *15*, 48, doi:10.1186/s12896-015-0168-2.
19. Sackmann, E.K.; Fulton, A.L.; Beebe, D.J. The present and future role of microfluidics in biomedical research. *Nature* **2014**, *507*, 181-189, doi:10.1038/nature13118.
20. Rodriguez-Bano, J.; Alcala, J.C.; Cisneros, J.M.; Grill, F.; Oliver, A.; Horcajada, J.P.; Tortola, T.; Mirelis, B.; Navarro, G.; Cuenca, M., et al. Community infections caused by extended-spectrum beta-lactamase-producing Escherichia coli. *Arch Intern Med* **2008**, *168*, 1897-1902, doi:10.1001/archinte.168.17.1897.
21. Morgand, M.; Vimont, S.; Bleibtreu, A.; Boyd, A.; Thien, H.V.; Zahar, J.-R.; Denamur, E.; Arlet, G. Extended-spectrum beta-lactamase-producing Escherichia coli infections in children: Are community-acquired strains different from nosocomial strains? *International Journal of Medical Microbiology* **2014**, *304*, 970-976, doi:https://doi.org/10.1016/j.ijmm.2014.06.003.
22. Lim, J.Y.; Yoon, J.W.; Hovde, C.J. A Brief Overview of Escherichia coli O157:H7 and Its Plasmid O157. *Journal of microbiology and biotechnology* **2010**, *20*, 5-14.
23. Ch. Schröder, U.; Beleites, C.; Assmann, C.; Glaser, U.; Hübner, U.; Pfister, W.; Fritzsche, W.; Popp, J.; Neugebauer, U. Detection of vancomycin resistances in enterococci within 3 ½ hours. *Scientific Reports* **2015**, *5*, 8217, doi:10.1038/srep08217 https://www.nature.com/articles/srep08217#supplementary-information.
24. Hrabak, J.; Chudackova, E.; Walkova, R. Matrix-assisted laser desorption ionization-time of flight (maldi-tof) mass spectrometry for detection of antibiotic resistance mechanisms: from research to routine diagnosis. *Clin Microbiol Rev* **2013**, *26*, 103-114, doi:10.1128/cmr.00058-12.
25. Lupo, A.; Papp-Wallace, K.M.; Sendi, P.; Bonomo, R.A.; Endimiani, A. Non-phenotypic tests to detect and characterize antibiotic resistance mechanisms in Enterobacteriaceae. *Diagn Microbiol Infect Dis* **2013**, *77*, 179-194, doi:10.1016/j.diagmicrobio.2013.06.001.

26. Pulido, M.R.; Garcia-Quintanilla, M.; Martin-Pena, R.; Cisneros, J.M.; McConnell, M.J. Progress on the development of rapid methods for antimicrobial susceptibility testing. *J Antimicrob Chemother* **2013**, *68*, 2710-2717, doi:10.1093/jac/dkt253.
27. Wiegand, I.; Hilpert, K.; Hancock, R.E. Agar and broth dilution methods to determine the minimal inhibitory concentration (MIC) of antimicrobial substances. *Nat Protoc* **2008**, *3*, 163-175, doi:10.1038/nprot.2007.521.
28. Chewning, J.H. Vancomycin-resistant Enterococcus faecium Bacteremia Successfully Treated With High-dose Ampicillin-Sulbactam in a Pediatric Patient After Hematopoietic Stem Cell Transplantation. *Journal of Pediatric Hematology/Oncology* **2011**, *33*, 401, doi:10.1097/MPH.0b013e31820db7eb.
29. O'Driscoll, T.; Crank, C.W. Vancomycin-resistant enterococcal infections: epidemiology, clinical manifestations, and optimal management. *Infection and drug resistance* **2015**, *8*, 217-230, doi:10.2147/IDR.S54125.
30. Kohler, P.; Eshaghi, A.; Kim, H.C.; Plevneshi, A.; Green, K.; Willey, B.M.; McGeer, A.; Patel, S.N. Prevalence of vancomycin-variable Enterococcus faecium (VVE) among vanA-positive sterile site isolates and patient factors associated with VVE bacteremia. *PLoS One* **2018**, *13*, e0193926, doi:10.1371/journal.pone.0193926.
31. Xu, J.; Moore, J.E.; Murphy, P.G.; Millar, B.C.; Elborn, J.S. Early detection of Pseudomonas aeruginosa – comparison of conventional versus molecular (PCR) detection directly from adult patients with cystic fibrosis (CF). *Annals of Clinical Microbiology and Antimicrobials* **2004**, *3*, 21-21, doi:10.1186/1476-0711-3-21.
32. Khan, A.A.; Cerniglia, C.E. Detection of Pseudomonas aeruginosa from clinical and environmental samples by amplification of the exotoxin A gene using PCR. *Applied and Environmental Microbiology* **1994**, *60*, 3739-3745.
33. Counts, G.W.; Schwartz, R.W.; Ulness, B.K.; Hamilton, D.J.; Rosok, M.J.; Cunningham, M.D.; Tam, M.R.; Darveau, R.P. Evaluation of an immunofluorescent-antibody test for rapid identification of Pseudomonas aeruginosa in blood cultures. *J Clin Microbiol* **1988**, *26*, 1161-1165.
34. Hachem, R.Y.; Chemaly, R.F.; Ahmar, C.A.; Jiang, Y.; Boktour, M.R.; Rjaili, G.A.; Bodey, G.P.; Raad, I.I. Colistin Is Effective in Treatment of Infections Caused by Multidrug-Resistant Pseudomonas aeruginosa in Cancer Patients. *Antimicrobial Agents and Chemotherapy* **2007**, *51*, 1905-1911, doi:10.1128/AAC.01015-06.
35. Tan, J.S.; File, T.M., Jr. Antipseudomonal penicillins. *Med Clin North Am* **1995**, *79*, 679-693.
36. Palavutitotai, N.; Jitmuang, A.; Tongsai, S.; Kiratisin, P.; Angkasekwinai, N. Epidemiology and risk factors of extensively drug-resistant Pseudomonas aeruginosa infections. *PLoS One* **2018**, *13*, e0193431-e0193431, doi:10.1371/journal.pone.0193431.
37. Khuntayaporn, P.; Montakantikul, P.; Santanirand, P.; Kiratisin, P.; Chomnawang, M.T. Molecular investigation of carbapenem resistance among multidrug-resistant Pseudomonas aeruginosa isolated clinically in Thailand. *Microbiol. Immunol.* **2013**, *57*, 170-178, doi:10.1111/1348-0421.12021.
38. Gupta, A.; Ampofo, K.; Rubenstein, D.; Saiman, L. Extended Spectrum β Lactamase-producing Klebsiella pneumoniae Infections: a Review of the Literature. *Journal Of Perinatology* **2003**, *23*, 439, doi:10.1038/sj.jp.7210973.
39. Sekowska, A.; Janicka, G.; Klyszejko, C.; Wojda, M.; Wroblewski, M.; Szymankiewicz, M. Resistance of Klebsiella pneumoniae strains producing and not producing ESBL (extended-spectrum beta-lactamase) type enzymes to selected non-beta-lactam antibiotics. *Med Sci Monit* **2002**, *8*, Br100-104.
40. Lee, C.H.; Su, L.H.; Tang, Y.F.; Liu, J.W. Treatment of ESBL-producing Klebsiella pneumoniae bacteraemia with carbapenems or flomoxef: a retrospective study and laboratory analysis of the isolates. *J Antimicrob Chemother* **2006**, *58*, 1074-1077, doi:10.1093/jac/dkl381.

41. Metri, B.C.; Jyothi, P.; Peerapur, B.V. Detection of ESBL in E.coli and K. pneumoniae isolated from urinary tract infection. *Indian Journal of Nephrology* **2012**, *22*, 401-402, doi:10.4103/0971-4065.103919.
42. Ghasemi, Y.; Archin, T.; Kargar, M.; Mohkam, M. A simple multiplex PCR for assessing prevalence of extended-spectrum β-lactamases producing Klebsiella pneumoniae in Intensive Care Units of a referral hospital in Shiraz, Iran. *Asian Pacific Journal of Tropical Medicine* **2013**, *6*, 703-708, doi:https://doi.org/10.1016/S1995-7645(13)60122-4.
43. Goble , M.; Iseman , M.D.; Madsen , L.A.; Waite , D.; Ackerson , L.; Horsburgh , C.R.J. Treatment of 171 Patients with Pulmonary Tuberculosis Resistant to Isoniazid and Rifampin. *New England Journal of Medicine* **1993**, *328*, 527-532, doi:10.1056/nejm199302253280802.
44. Ziganshina, L.E.; Vizel, A.A.; Squire, S.B. Fluoroquinolones for treating tuberculosis. *Cochrane Database of Systematic Reviews* **2005**, 10.1002/14651858.CD004795.pub2, doi:10.1002/14651858.CD004795.pub2.
45. Mitnick, C.D.; Appleton, S.C.; Shin, S.S. Epidemiology and Treatment of Multidrug Resistant Tuberculosis. *Seminars in respiratory and critical care medicine* **2008**, *29*, 499-524, doi:10.1055/s-0028-1085702.
46. Pigrau-Serrallach, C.; Rodríguez-Pardo, D. Bone and joint tuberculosis. *European Spine Journal* **2013**, *22*, 556-566, doi:10.1007/s00586-012-2331-y.
47. Choi, W.; Lee, J.; Cho, E.; Jung, G.Y. Accurate and effective multidrug-resistant Mycobacterium tuberculosis detection method using gap-filling ligation coupled with high-resolution capillary electrophoresis-based single strand conformation polymorphism. *Scientific Reports* **2017**, *7*, 46090, doi:10.1038/srep46090.
48. Ormerod, L.P. Multidrug-resistant tuberculosis (MDR-TB): epidemiology, prevention and treatment. *Br. Med. Bull.* **2005**, *73-74*, 17-24, doi:10.1093/bmb/ldh047.
49. Howard, A.; O'Donoghue, M.; Feeney, A.; Sleator, R.D. Acinetobacter baumannii: An emerging opportunistic pathogen. *Virulence* **2012**, *3*, 243-250, doi:10.4161/viru.19700.
50. Guerrero, D.M.; Perez, F.; Conger, N.G.; Solomkin, J.S.; Adams, M.D.; Rather, P.N.; Bonomo, R.A. Acinetobacter baumannii-Associated Skin and Soft Tissue Infections: Recognizing a Broadening Spectrum of Disease. *Surgical Infections* **2010**, *11*, 49-57, doi:10.1089/sur.2009.022.
51. Li, P.; Niu, W.k.; Li, H.; Lei, H.; Liu, W.; Zhao, X.; Guo, L.; Zou, D.; Yuan, X.; Liu, H., et al. Rapid detection of Acinetobacter baumannii and molecular epidemiology of carbapenem-resistant A. baumannii in two comprehensive hospitals of Beijing, China. *Frontiers in Microbiology* **2015**, *6*, doi:10.3389/fmicb.2015.00997.
52. Turton, J.F.; Woodford, N.; Glover, J.; Yarde, S.; Kaufmann, M.E.; Pitt, T.L. Identification of Acinetobacter baumannii by Detection of the bla(OXA-51-like) Carbapenemase Gene Intrinsic to This Species. *Journal of Clinical Microbiology* **2006**, *44*, 2974-2976, doi:10.1128/JCM.01021-06.
53. Suwantarat, N.; Carroll, K.C. Epidemiology and molecular characterization of multidrug-resistant Gram-negative bacteria in Southeast Asia. *Antimicrobial resistance and infection control* **2016**, *5*, 15-15, doi:10.1186/s13756-016-0115-6.
54. Rogers, K.L.; Fey, P.D.; Rupp, M.E. Coagulase-negative staphylococcal infections. *Infect Dis Clin North Am* **2009**, *23*, 73-98, doi:10.1016/j.idc.2008.10.001.
55. Otto, M. Staphylococcus epidermidis – the "accidental" pathogen. *Nature reviews. Microbiology* **2009**, *7*, 555-567, doi:10.1038/nrmicro2182.
56. Ikeda, Y.; Ohara-Nemoto, Y.; Kimura, S.; Ishibashi, K.; Kikuchi, K. PCR-based identification of Staphylococcus epidermidis targeting gseA encoding the glutamic-acid-specific protease. *Canadian Journal of Microbiology* **2004**, *50*, 493-498, doi:10.1139/w04-055.
57. Chessa, D.; Ganau, G.; Mazzarello, V. An overview of Staphylococcus epidermidis and Staphylococcus aureus with a focus on developing countries. *Journal of infection in developing countries* **2015**, *9*, 547-550, doi:10.3855/jidc.6923.

58. Poole, K. Efflux-Mediated Resistance to Fluoroquinolones in Gram-Negative Bacteria. *Antimicrobial Agents and Chemotherapy* **2000**, *44*, 2233-2241.
59. Zhang, L.; Li, X.-Z.; Poole, K. Fluoroquinolone susceptibilities of efflux-mediated multidrug-resistant Pseudomonas aeruginosa, Stenotrophomonas maltophilia and Burkholderia cepacia. *Journal of Antimicrobial Chemotherapy* **2001**, *48*, 549-552, doi:10.1093/jac/48.4.549.
60. Horsley, A.; Jones, A.M.; Lord, R. Antibiotic treatment for Burkholderia cepacia complex in people with cystic fibrosis experiencing a pulmonary exacerbation. *Cochrane Database of Systematic Reviews* **2016**, 10.1002/14651858.CD009529.pub3, doi:10.1002/14651858.CD009529.pub3.
61. Aaron, S.D.; Ferris, W.; Henry, D.A.; Speert, D.P.; Macdonald, N.E. Multiple combination bactericidal antibiotic testing for patients with cystic fibrosis infected with Burkholderia cepacia. *Am J Respir Crit Care Med* **2000**, *161*, 1206-1212, doi:10.1164/ajrccm.161.4.9907147.
62. Mukhopadhyay, C.; Bhargava, A.; Ayyagari, A. Two novel clinical presentations of Burkholderia cepacia infection. *J Clin Microbiol* **2004**, *42*, 3904-3905, doi:10.1128/jcm.42.8.3904-3905.2004.
63. McDowell, A.; Mahenthiralingam, E.; Moore, J.E.; Dunbar, K.E.; Webb, A.K.; Dodd, M.E.; Martin, S.L.; Millar, B.C.; Scott, C.J.; Crowe, M., et al. PCR-based detection and identification of Burkholderia cepacia complex pathogens in sputum from cystic fibrosis patients. *J Clin Microbiol* **2001**, *39*, 4247-4255, doi:10.1128/jcm.39.12.4247-4255.2001.
64. Spencer, R.C. The emergence of epidemic, multiple-antibiotic-resistant Stenotrophomonas (Xanthomonas) maltophilia and Burkholderia (Pseudomonas) cepacia. *J. Hosp. Infect.* **1995**, *30*, 453-464, doi:https://doi.org/10.1016/0195-6701(95)90049-7.
65. Geraats-Peters, C.W.M.; Brouwers, M.; Schneeberger, P.M.; van der Zanden, A.G.M.; Bruisten, S.M.; Weers-Pothoff, G.; Boel, C.H.E.; van den Brule, A.J.C.; Harmsen, H.G.; Hermans, M.H.A. Specific and Sensitive Detection of Neisseria gonorrhoeae in Clinical Specimens by Real-Time PCR. *Journal of Clinical Microbiology* **2005**, *43*, 5653-5659, doi:10.1128/JCM.43.11.5653-5659.2005.
66. Ng, L.-K.; Martin, I.E. The laboratory diagnosis of Neisseria gonorrhoeae. *The Canadian Journal of Infectious Diseases & Medical Microbiology* **2005**, *16*, 15-25.
67. Yoldaş, Ö.; Altındiş, M.; Cufalı, D.; Aşık, G.; Keşli, R. A Diagnostic Algorithm for the Detection of Clostridium difficile-Associated Diarrhea. *Balkan Medical Journal* **2016**, *33*, 80-86, doi:10.5152/balkanmedj.2015.15159.
68. Huang, H.; Weintraub, A.; Fang, H.; Nord, C.E. Antimicrobial resistance in Clostridium difficile. *International Journal of Antimicrobial Agents* **2009**, *34*, 516-522, doi:https://doi.org/10.1016/j.ijantimicag.2009.09.012.
69. Spigaglia, P. Recent advances in the understanding of antibiotic resistance in Clostridium difficile infection. *Therapeutic Advances in Infectious Disease* **2016**, *3*, 23-42, doi:10.1177/2049936115622891.
70. Balsells, E.; Shi, T.; Leese, C.; Lyell, I.; Burrows, J.; Wiuff, C.; Campbell, H.; Kyaw, M.H.; Nair, H. Global burden of Clostridium difficile infections: a systematic review and meta-analysis. *Journal of global health* **2019**, *9*, 010407-010407, doi:10.7189/jogh.09.010407.
71. Delignette-Muller, M.; Flandrois, J. An accurate diffusion method for determining bacterial sensitivity to antibiotics. *Journal of antimicrobial chemotherapy* **1994**, *34*, 73-81.
72. McDermott, P.F.; Bodeis-Jones, S.M.; Fritsche, T.R.; Jones, R.N.; Walker, R.D. Broth microdilution susceptibility testing of Campylobacter jejuni and the determination of quality control ranges for fourteen antimicrobial agents. *Journal of clinical microbiology* **2005**, *43*, 6136-6138.
73. Murray, C.K.; Hospenthal, D.R. Broth microdilution susceptibility testing for Leptospira spp. *Antimicrobial agents and chemotherapy* **2004**, *48*, 1548-1552.

74. Miller, R.A.; Walker, R.D.; Carson, J.; Coles, M.; Coyne, R.; Dalsgaard, I.; Gieseker, C.; Hsu, H.M.; Mathers, J.J.; Papapetropoulou, M., et al. Standardization of a broth microdilution susceptibility testing method to determine minimum inhibitory concentrations of aquatic bacteria. *Diseases of aquatic organisms* **2005**, *64*, 211-222, doi:10.3354/dao064211.
75. Waites, K.B.; Duffy, L.B.; Bébéar, C.M.; Matlow, A.; Talkington, D.F.; Kenny, G.E.; Totten, P.A.; Bade, D.J.; Zheng, X.; Davidson, M.K. Standardized methods and quality control limits for agar and broth microdilution susceptibility testing of Mycoplasma pneumoniae, Mycoplasma hominis, and Ureaplasma urealyticum. *Journal of clinical microbiology* **2012**, JCM. 01439-01412.
76. Tomida, J.; Oumi, A.; Okamoto, T.; Morita, Y.; Okayama, A.; Misawa, N.; Hayashi, T.; Akaike, T.; Kawamura, Y. Comparative evaluation of agar dilution and broth microdilution methods for antibiotic susceptibility testing of Helicobacter cinaedi. *Microbiology and immunology* **2013**, *57*, 353-358, doi:10.1111/1348-0421.12044.
77. Siddiqi, S.H.; Heifets, L.B.; Cynamon, M.H.; Hooper, N.M.; Laszlo, A.; Libonati, J.P.; Lindholm-Levy, P.J.; Pearson, N. Rapid broth macrodilution method for determination of MICs for Mycobacterium avium isolates. *J Clin Microbiol* **1993**, *31*, 2332-2338.
78. Glupczynski, Y.; Labbe, M.; Hansen, W.; Crokaert, F.; Yourassowsky, E. Evaluation of the E test for quantitative antimicrobial susceptibility testing of Helicobacter pylori. *Journal of Clinical Microbiology* **1991**, *29*, 2072-2075.
79. Brown, D.F.; Brown, L. Evaluation of the E test, a novel method of quantifying antimicrobial activity. *The Journal of antimicrobial chemotherapy* **1991**, *27*, 185-190.
80. Baker, C.N. The E-Test and Campylobacter jejuni. *Diagnostic microbiology and infectious disease* **1992**, *15*, 469-472.
81. Schulz, J.; Sahm, D. Reliability of the E test for detection of ampicillin, vancomycin, and high-level aminoglycoside resistance in Enterococcus spp. *Journal of clinical microbiology* **1993**, *31*, 3336-3339.
82. Cantón, R.; Livermore, D.M.; Morosini, M.I.; Díaz-Regañón, J.; Rossolini, G.M.; Group, P.S.; Group, P.S. Etest® versus broth microdilution for ceftaroline MIC determination with Staphylococcus aureus: results from PREMIUM, a European multicentre study. *Journal of Antimicrobial Chemotherapy* **2016**, dkw442.
83. Clark, A.E.; Kaleta, E.J.; Arora, A.; Wolk, D.M. Matrix-assisted laser desorption ionization–time of flight mass spectrometry: a fundamental shift in the routine practice of clinical microbiology. *Clinical microbiology reviews* **2013**, *26*, 547-603.
84. Singhal, N.; Kumar, M.; Kanaujia, P.K.; Virdi, J.S. MALDI-TOF mass spectrometry: an emerging technology for microbial identification and diagnosis. *Frontiers in Microbiology* **2015**, *6*, doi:10.3389/fmicb.2015.00791.
85. Tré-Hardy, M.; Lambert, B.; Despas, N.; Bressant, F.; Laurenzano, C.; Rodriguez-Villalobos, H.; Verroken, A. MALDI-TOF MS identification and antimicrobial susceptibility testing directly from positive enrichment broth. *Journal of Microbiological Methods* **2017**, *141*, 32-34, doi:https://doi.org/10.1016/j.mimet.2017.07.012.
86. Strommenger, B.; Kettlitz, C.; Werner, G.; Witte, W. Multiplex PCR assay for simultaneous detection of nine clinically relevant antibiotic resistance genes in Staphylococcus aureus. *J Clin Microbiol* **2003**, *41*, 4089-4094.
87. Cockerill, F.R. Genetic methods for assessing antimicrobial resistance. *Antimicrobial agents and chemotherapy* **1999**, *43*, 199-212.
88. Strommenger, B.; Schmidt, C.; Werner, G.; Roessle-Lorch, B.; Bachmann, T.T.; Witte, W. DNA microarray for the detection of therapeutically relevant antibiotic resistance determinants in clinical isolates of Staphylococcus aureus. *Molecular and cellular probes* **2007**, *21*, 161-170, doi:10.1016/j.mcp.2006.10.003.
89. Miller, M.B.; Tang, Y.-W. Basic Concepts of Microarrays and Potential Applications in Clinical Microbiology. *Clinical Microbiology Reviews* **2009**, *22*, 611-633, doi:10.1128/CMR.00019-09.

90. Lu, N.; Hu, Y.; Zhu, L.; Yang, X.; Yin, Y.; Lei, F.; Zhu, Y.; Du, Q.; Wang, X.; Meng, Z., et al. DNA microarray analysis reveals that antibiotic resistance-gene diversity in human gut microbiota is age related. *Scientific Reports* **2014**, *4*, 4302, doi:10.1038/srep04302

https://www.nature.com/articles/srep04302#supplementary-information.

91. Bobenchik, A.M.; Hindler, J.A.; Giltner, C.L.; Saeki, S.; Humphries, R.M. Performance of Vitek 2 for Antimicrobial Susceptibility Testing of Staphylococcus spp. and Enterococcus spp. *Journal of Clinical Microbiology* **2014**, *52*, 392-397, doi:10.1128/JCM.02432-13.
92. van Belkum, A.; Dunne, W.M. Next-generation antimicrobial susceptibility testing. *Journal of clinical microbiology* **2013**, *51*, 2018-2024.
93. Pykett, A.H. An assessment of the Sensititre method for determining minimum inhibitory and bactericidal concentrations. *Journal of clinical pathology* **1978**, *31*, 536-538.
94. Doern, G.; Staneck, J.; Needham, C.; Tubert, T. Sensititre autoreader for same-day breakpoint broth microdilution susceptibility testing of members of the family Enterobacteriaceae. *Journal of clinical microbiology* **1987**, *25*, 1481-1485.
95. Staneck, J.L.; Allen, S.D.; Harris, E.E.; Tilton, R.C. Rapid MIC testing with the sensititre autoreader. *Journal of clinical microbiology* **1988**, *26*, 1-7.
96. Richter, S.S.; Ferraro, M.J. Susceptibility testing instrumentation and computerized expert systems for data analysis and interpretation. In *Manual of Clinical Microbiology, 10th Edition*, American Society of Microbiology: ASM Press, P.O. Box 605, Herndon, VA, USA 2011; pp. 1144-1154.
97. Jang, W.; Park, Y.-J.; Park, K.G.; Yu, J. Evaluation of MicroScan WalkAway and Vitek 2 for determination of the susceptibility of extended-spectrum β-lactamase-producing Escherichia coli and Klebsiella pneumoniae isolates to cefepime, cefotaxime and ceftazidime. *Journal of Antimicrobial Chemotherapy* **2013**, *68*, 2282-2285, doi:10.1093/jac/dkt172.
98. Burns, J.L.; Saiman, L.; Whittier, S.; Krzewinski, J.; Liu, Z.; Larone, D.; Marshall, S.A.; Jones, R.N. Comparison of two commercial systems (Vitek and MicroScan-WalkAway) for antimicrobial susceptibility testing of Pseudomonas aeruginosa isolates from cystic fibrosis patients. *Diagnostic microbiology and infectious disease* **2001**, *39*, 257-260.
99. Wellinghausen, N.; Pietzcker, T.; Poppert, S.; Belak, S.; Fieser, N.; Bartel, M.; Essig, A. Evaluation of the Merlin MICRONAUT System for Rapid Direct Susceptibility Testing of Gram-Positive Cocci and Gram-Negative Bacilli from Positive Blood Cultures. *Journal of Clinical Microbiology* **2007**, *45*, 789-795, doi:10.1128/jcm.01856-06.
100. Wright, D.N.; Matsen, J.M.; DiPersio, J.R.; Kirk, M.; Saxon, B.; Ficorilli, S.M.; Spencer, H.J. Evaluation of four newer antimicrobial agents in the Avantage susceptibility test system. *Journal of Clinical Microbiology* **1989**, *27*, 2381-2383.
101. Nolte, F.S.; Contestable, P.B.; Lincalis, D.; Punsalang Jr, A. Rapid, direct antibiotic susceptibility testing of blood culture isolates using the Abbott avantage® system. *American journal of clinical pathology* **1986**, *86*, 665-669.
102. Sriboonsong, S.; Boonchoo, L. Automatic antimicrobial susceptibility testing system. In Proceedings of World Congress on Medical Physics and Biomedical Engineering 2006; pp. 2501-2504.
103. Golchin, S.A.; Stratford, J.; Curry, R.J.; McFadden, J. A microfluidic system for long-term time-lapse microscopy studies of mycobacteria. *Tuberculosis* **2012**, *92*, 489-496.
104. Mohan, R.; Mukherjee, A.; Sevgen, S.E.; Sanpitakseree, C.; Lee, J.; Schroeder, C.M.; Kenis, P.J.A. A multiplexed microfluidic platform for rapid antibiotic susceptibility testing. *Biosensors and Bioelectronics* **2013**, *49*, 118-125.
105. Mohan, R.; Sanpitakseree, C.; Desai, A.V.; Sevgen, S.E.; Schroeder, C.M.; Kenis, P.J.A. A microfluidic approach to study the effect of bacterial interactions on antimicrobial susceptibility in polymicrobial cultures. *RSC Adv.* **2015**, *5*, 35211-35223, doi:10.1039/C5RA04092B.

106. Webster, T.A.; Sismaet, H.J.; Chan, I.p.J.; Goluch, E.D. Electrochemically monitoring the antibiotic susceptibility of Pseudomonas aeruginosa biofilms. *Analyst* **2015**, *140*, 7195-7201, doi:10.1039/C5AN01358E.
107. Baker, C.A.; Duong, C.T.; Grimley, A.; Roper, M.G. Recent advances in microfluidic detection systems. *Bioanalysis* **2009**, *1*, 967-975, doi:10.4155/bio.09.86.
108. Wang, Y.; Xu, H.; Zhang, J.; Li, G. Electrochemical Sensors for Clinic Analysis. *Sensors (Basel, Switzerland)* **2008**, *8*, 2043-2081.
